# Temporal Transcriptome Analysis Reveals Core Pathways and Orphan Gene *EARLY FLOWERING 1* Regulating Floral Transition in Chinese Cabbage

**DOI:** 10.3390/plants14142236

**Published:** 2025-07-19

**Authors:** Hong Lang, Yuting Zhang, Shouhe Zhao, Kexin Li, Xiaonan Li, Mingliang Jiang

**Affiliations:** 1 School of Agriculture, Jilin Agricultural Science and Technology College, Jilin 132101, China; langhong@jlnku.edu.cn (H.L.); likexin0512@163.com (K.L.); 2 Molecular Biology of Vegetable Laboratory, College of Horticulture, Shenyang Agricultural University, Shenyang 110866, China; zhangyuting@caas.cn (Y.Z.); zsh20021212@163.com (S.Z.); 3 Institute of Vegetables and Flowers, Chinese Academy of Agricultural Sciences, Beijing 100081, China

**Keywords:** Chinese cabbage, floral transition, transcriptome profiling, hormone signaling, MAPK signaling, orphan genes, *EARLY FLOWERING 1*

## Abstract

The floral transition in Chinese cabbage (*Brassica rapa* ssp. *pekinensis*) is governed by a complex interplay of gene expression and hormonal regulation. Temporal transcriptome profiling was conducted across three developmental stages: pre-bolting (PBS), bolting (BS), and flowering stages (FS), to investigate the underlying molecular mechanisms. A total of 7092 differentially expressed genes (DEGs) were identified, exhibiting distinct expression trajectories during the transition. Moreover, functional enrichment analyses revealed strong associations with plant hormone signaling, MAPK pathways, and developmental regulation processes. Key flowering-related genes, such as *BrFLM*, *BrAP2*, *BrFD*, *BrFT*, and *BrSOC1s* displayed antagonistic expression patterns. Hormonal pathways involving auxin, ABA, ET, BR, GA, JA, CK, and SA showed stage-dependent modulation. Further, orphan genes (OGs), especially *EARLY FLOWERING 1* (*EF1*), showed significant upregulation during the transition, which exhibited 1.84-fold and 1.93-fold increases at BS and FS compared to PBS, respectively (*p* < 0.05). Functional validation through *EF1* overexpression (EF1OE) in *Arabidopsis* consistently promoted early flowering. The expression levels of *AtFT* and *AtSOC1* were significantly upregulated in EF1OE lines compared to wild-type (WT) plants. The findings contribute to understanding the coordinated genetic and hormonal events driving floral development in Chinese cabbage, suggesting *EF1* as a candidate for bolting resistance breeding. This work also expands the existing regulatory framework through the successful integration of OGs into the complex floral induction system of *Brassica* crops.

## 1. Introduction

Chinese cabbage (*Brassica rapa* ssp. *pekinensis*), is an important crop worldwide, classified under the Brassicaceae family, and has long been cultivated for both its economic benefits and nutritional content [1]. This crop forms a compact leafy head through the inward curling of the apical blades, creating overlapping layers during the rosette-to-heading transition [2]. With its rapid growth, high yield, and vitamin- and fiber-rich leaves, Chinese cabbage holds a central role among *Brassica* crops in Asia [3,4]. However, the incidence of premature bolting, especially during spring or at elevated altitudes, adversely affects yield and product quality by disrupting head formation, thus constraining suitable regions and growing seasons. This underscores the importance of identifying genetic determinants of bolting resistance genes and breeding bolting-resistant cultivars [5]. The premature bolting in Chinese cabbage is a complex physiological process regulated by the intricate interplay of hormonal signaling pathways, environmental factors (particularly temperature and photoperiod), and genetic determinants [2,6]. In light of the crop’s agronomic importance and the intricate regulatory factors involved, clarifying the molecular mechanisms governing floral transition is essential to breeding cultivars that resist bolting while preserving yield and quality standards.

Floral transition represents a genetically regulated developmental switch that shifts shoot apical meristems from vegetative to reproductive growth, marking a crucial life-history phase with direct implications for plant reproductive success and fitness [7,8]. Recent advances in floral transition research have identified five core flowering regulation pathways: the age, vernalization, autonomous, photoperiod, and gibberellin (GA) pathways [9,10,11,12,13,14]. Moreover, novel regulatory factors, such as light intensity, thermosensory, sugar availability, abiotic stress, CO_2_ levels, and hormonal signals, have been identified as contributors to floral transition alongside established genetic networks [15,16,17]. These pathways converge through a core group of floral pathway integrators, including *FLOWERING LOCUS C* (*FLC*), *FLOWERING LOCUS T* (*FT*), *SUPPRESSOR OF OVEREXPRESSION OF CONSTANS 1* (*SOC1*), *LEAFY* (*LFY*), *APETALA1* (*AP1*), and *AGAMOUS-LIKE 24* (*AGL24*), which determine the flowering time by coordinating developmental and environmental cues [18,19,20]. The functional analysis of flowering-related genes, including *FRIGIDA* (*BrFRIs*) [21], *BrFLCs* [22,23,24,25], *BrSOCs* [26], *BrFTs* [27,28], *CURLY LEAF* (*BrCLF*) [29], *SET DOMAIN GROUP 8* (*BrSDG8*) [30], and *ent-kaurene synthase* (*BrKS*) [31], has provided a basis for developing targeted breeding strategies to manipulate the flowering time and expand germplasm diversity in Chinese cabbage. Although substantial progress has been achieved in the characterization of flowering pathways in Chinese cabbage, the potential regulatory functions of orphan genes (OGs) in floral transition remain fundamentally unexplored.

OGs, distinguished by the lack of sequence homology to genes in other lineages, have been identified across multiple plant species including *B. rapa* [32]. While the functional characterization of most OGs remains incomplete, emerging evidence indicates their participation in core metabolic pathways, environmental stress adaptation, and the evolution of species-specific traits [2]. The rice-specific OG *OsFON879*, which lacks conserved domains, plays a vital role in floral organ development by interacting with OsRRM1, connecting OG function to the RNA metabolic control of floral meristem networks [33]. In bread wheat, male fertility and microgametogenesis depend on the Poaceae-specific gene *Male Sterility 1* (*Ms1*) [34], and the Triticinae-specific gene *Male Sterility 2* (*Ms2*) confers male sterility across wheat, barley, and *Brachypodium* species [35]. Recent studies have revealed an OG-mediated flowering regulation pathway in Chinese cabbage, including *BOLTING RESISTANCE 1* (*BR1*), *BOLTING RESISTANCE 2* (*BR2*), *BOLTING RESISTANCE 3* (*BR3*), and *BOLTING RESISTANCE 4* (*BR4*), representing a breakthrough in understanding floral transition [5]. Furthermore, the screening of the *B. rapa* OGs (BrOGs) overexpression library in *Arabidopsis* revealed several unique regulators of the flowering time, with transgenic lines exhibiting substantial temporal variation in floral transition, ranging from marked delays to accelerated flowering compared to wild-type plants [2]. Collectively, these findings underscore the expanding regulatory significance of OGs in modulating floral transition and broader developmental processes in plants. However, their precise temporal expression dynamics and regulatory functions during floral transition in Chinese cabbage are still largely unexplored.

To address the existing knowledge gaps, temporal transcriptome profiling was conducted across three key developmental stages, pre-bolting, bolting, and flowering stages, to analyze the dynamic expression patterns of both core flowering pathway genes and OGs, with selected candidates further validated through qRT-PCR. Particularly, functional characterization of the OG *EARLY FLOWERING 1* (*EF1*) revealed its promotive role in floral initiation, as evidenced by heterologous overexpression in *Arabidopsis*. The discovery of *EF1* establishes a promising molecular target for the precise regulation of the flowering time in Chinese cabbage. This study offers a model for understanding temporal gene regulation during floral transition in Chinese cabbage and highlights the potential of OGs as a new research direction for genetic improvement in crop breeding.

## 2. Results

### 2.1. Temporal Transcriptome Profiling of Floral Transition in Chinese Cabbage

**Figure 1:**
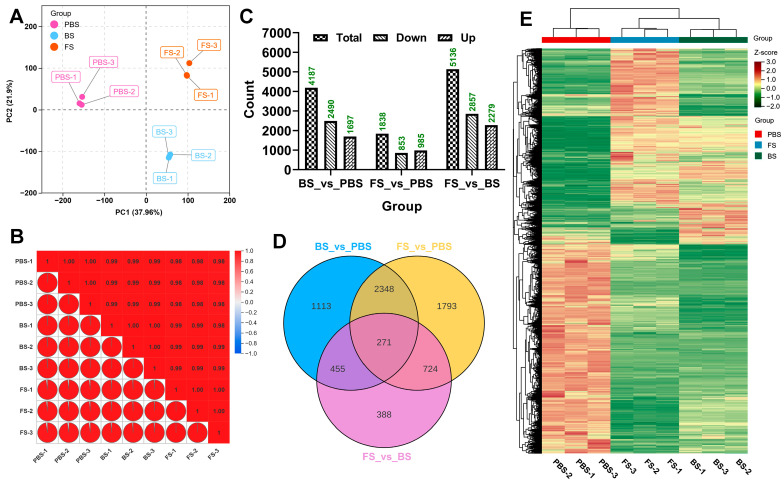
Temporal transcriptome dynamics during floral transition in Chinese cabbage. (**A**) Principal component analysis (PCA) plot. (**B**) Correlation analysis across samples. Red indicates positive correlation; blue indicates negative correlation. (**C**) The count of significantly upregulated and downregulated genes across different groups. (**D**) Comparative Venn analysis of DEGs. Blue represents BS_vs_PBS group; yellow represents FS_vs_PBS group; pink represents FS_vs_BS group. (**E**) Heat map analysis. Red represents upregulated genes; green represents downregulated genes. PBS, BS, and FS represent the pre-bolting stage, bolting stage, and flowering stage, respectively.

Transcriptomic sequencing of leaf tissues from the PBS, BS, and FS stages of Chinese cabbage was conducted to investigate dynamic gene expression changes during floral transition. High-throughput sequencing generated high-quality data across the three developmental stages, with Q20 > 97% and Q30 > 93% in all samples. Each library produced between 41.25 and 48.59 million raw reads, with 39.45 to 46.70 million clean reads obtained following quality filtering (Table S1). Moreover, clean reads of high quality were aligned to the *B. rapa* v2.5 reference genome, resulting in mapping efficiencies ranging from 92.5% to 93.2% across all samples (Table S2). A principal component analysis (PCA) conducted on FPKM data revealed distinct sample segregation, with significant intra-group clustering evident in the two-dimensional space (Figure 1A). A correlation analysis demonstrated high reproducibility across biological replicates, with all pairwise comparisons showing strong concordance (Figure 1B). Analysis revealed 1697, 985, and 2279 significantly upregulated differentially expressed genes (DEGs), along with 2490, 853, and 2857 significantly downregulated DEGs in the BS_vs_PBS, FS_vs_PBS, and FS_vs_BS comparisons, respectively (Figure 1C). The FS_vs_BS comparison exhibited the most marked transcriptomic changes. A set of 271 DEGs was consistently conserved and regulated across all three developmental transitions, while a stage-specific analysis identified 1113 unique DEGs in BS_vs_PBS, 1793 in FS_vs_PBS, and 388 in FS_vs_BS (Figure 1D). In total, 7092 DEGs were identified across all comparisons, each exhibiting a distinct temporal expression profile associated with specific stages of development (Figure 1E). These transcriptomic findings demonstrate stage-dependent gene expression dynamics during floral transition in Chinese cabbage, identifying 7092 DEGs with distinct expression patterns across vegetative-to-reproductive stages.

### 2.2. GO Classification and KEGG Pathway Enrichment of DEGs

**Figure 2:**
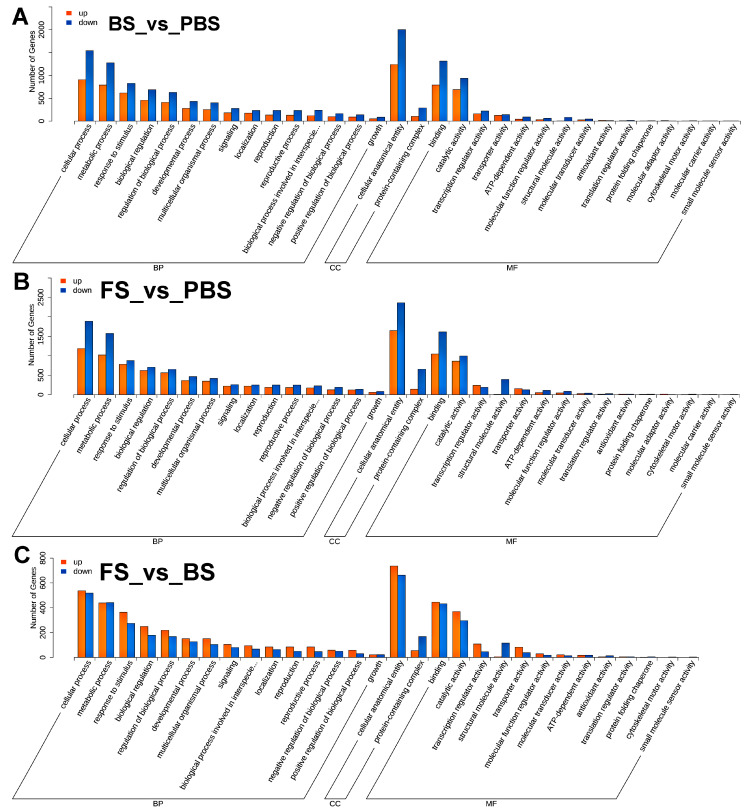
Go classification analysis of DEGs during floral transition in Chinese cabbage. (**A**) BS_vs_PBS group. (**B**) FS_vs_PBS group. (**C**) FS_vs_BS group. BP, CC, and MF indicate biological process, cellular component, and molecular function, respectively. The orange and blue bar charts represent upregulated and downregulated genes, respectively. PBS, BS, and FS represent the pre-bolting stage, bolting stage, and flowering stage, respectively. The horizontal axis of BP terms with ellipsis represents the biological process involved in interspecies interaction between organisms.

**Figure 3:**
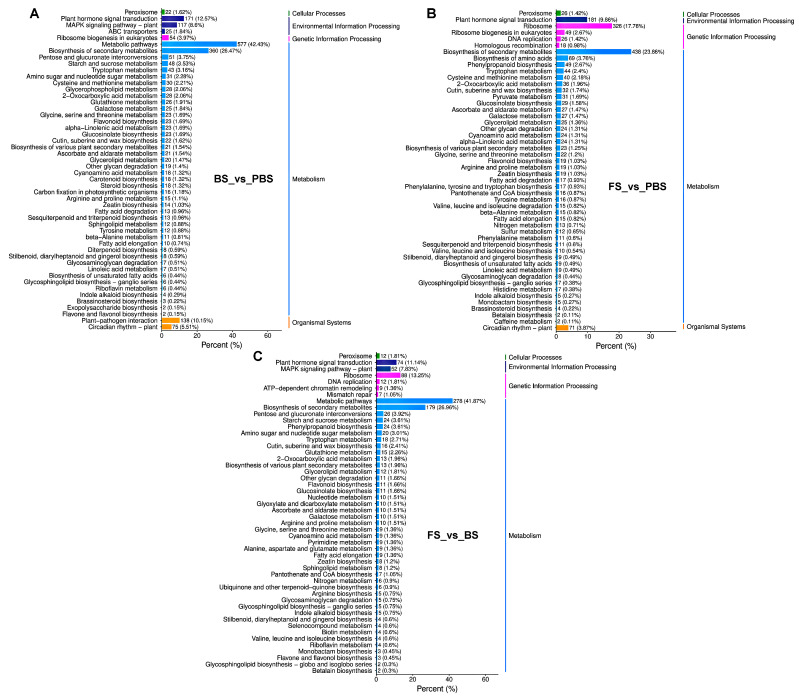
KEGG pathway enrichment analysis of DEGs during floral transition in Chinese cabbage. (**A**) BS_vs_PBS group. (**B**) FS_vs_PBS group. (**C**) FS_vs_BS group. The green, purple, pink, blue, and yellow bar charts respectively represent cellular processes, environmental information processing, genetic information processing, metabolism, and organismal systems. PBS, BS, and FS represent the pre-bolting stage, bolting stage, and flowering stage, respectively.

Analysis using KEGG pathway enrichment and GO classification was performed to describe the functional characteristics of the discovered DEGs. These DEGs were categorized by GO analysis into three categories: molecular function (MF), cellular component (CC), and biological process (BP) (Figure 2). Moreover, enriched BP terms included cellular process, metabolic process, response to stimulus, biological regulation, regulation of biological process, developmental process, multicellular organismal process, signaling, reproduction, and reproductive process. Among the CC terms, cellular anatomical entity and protein-containing complex were the most significantly enriched functional categories. Meanwhile, the MF category was enriched for binding, catalytic activity, and transcription regulator activity. The KEGG pathway analysis revealed a significant enrichment of DEGs in plant hormone signal transduction, MAPK signaling pathway–plant, general metabolic pathways, and the biosynthesis of secondary metabolites (Figure 3). Functional studies identified DEGs mostly enriched in developmental regulation, cellular architecture, molecular interactions, and hormone and MAPK signaling pathways during floral transition.

### 2.3. Transcriptional Dynamics of Core Flowering Pathway DEGs

**Figure 4:**
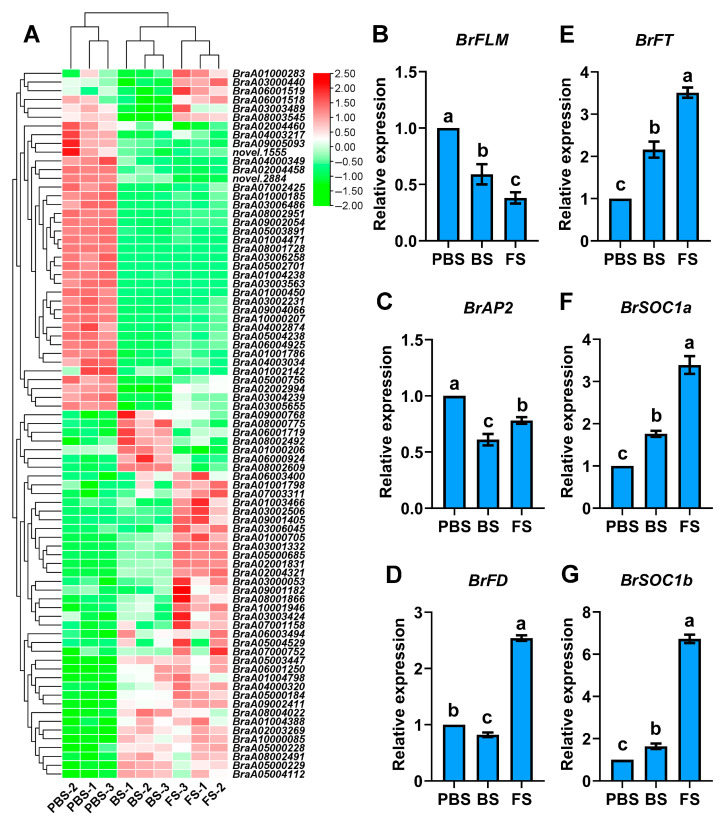
Expression patterns of core flowering pathway DEGs. (**A**) Heatmap analysis. Each column denotes an independent biological sample, while each row represents a unique gene transcript. Red represents upregulated genes; green represents downregulated genes. (**B**–**G**) qRT-PCR validation. Different lowercase letters denote statistically significant differences (*p* < 0.05, one-way ANOVA). PBS, BS, and FS represent the pre-bolting stage, bolting stage, and flowering stage, respectively.

A comparative analysis of critical DEGs associated with the floral transition pathway was conducted across various developmental comparisons. In total, 81 core DEGs were identified, comprising 35 downregulated and 24 upregulated genes in BS_vs_PBS, 27 downregulated and 32 upregulated in FS_vs_PBS, and 5 downregulated and 19 upregulated in FS_vs_BS (Figure 4A and Table S3). These findings uncovered a tightly coordinated regulatory network marked by a strong downregulation of floral repressors and concurrent upregulation of floral activators throughout the floral transition process in Chinese cabbage. During the vegetative-to-reproductive transition, the major repressors included *FLOWERING LOCUS M* (*BrFLM*, *BraA02004458*), *APETALA 2* (*BrAP2*, *BraA01000185*), *EARLY FLOWERING 4* (*BrELF4*, *BraA03002231*), *AGAMOUS-LIKE 18* (*BrAGL18*, *BraA04000349*), while the principal activators consisted of *Flowering Locus D* (*BrFD*, *BraA01000283*), *FT* (*BrFT*, *BraA02001831*), *SOC1s* (*BrSOC1a*, *BraA03002506*; *BrSOC1b*, *BraA05000685*), *AGAMOUS-LIKE 42* (*BrAGL42*, *BraA09000768*), *FLOWERING-PROMOTING FACTOR 1-LIKE PROTEIN 1* (*BrFLP1*, *BraA08001866*), and *SQUAMOSA PROMOTER-BINDING PROTEIN-LIKE 15* (*SPL15*, *BraA04000320*). The transcriptomic data were validated through qRT-PCR analysis of four randomly chosen genes. The relative expression levels of *BrFLM*, *BrAP2*, *BrFD*, *BrFT*, and *BrSOC1s* during floral transition were consistent with transcriptomic profiling results, confirming the observed expression trends (Figure 4B–G). These results reveal a dynamic transcriptional network in Chinese cabbage, where precisely orchestrated antagonistic interactions between flowering activators and repressors fine-tune the transition to reproductive development.

### 2.4. Transcriptional Profiling of DEGs in Plant Hormone Signal Transduction

**Figure 5:**
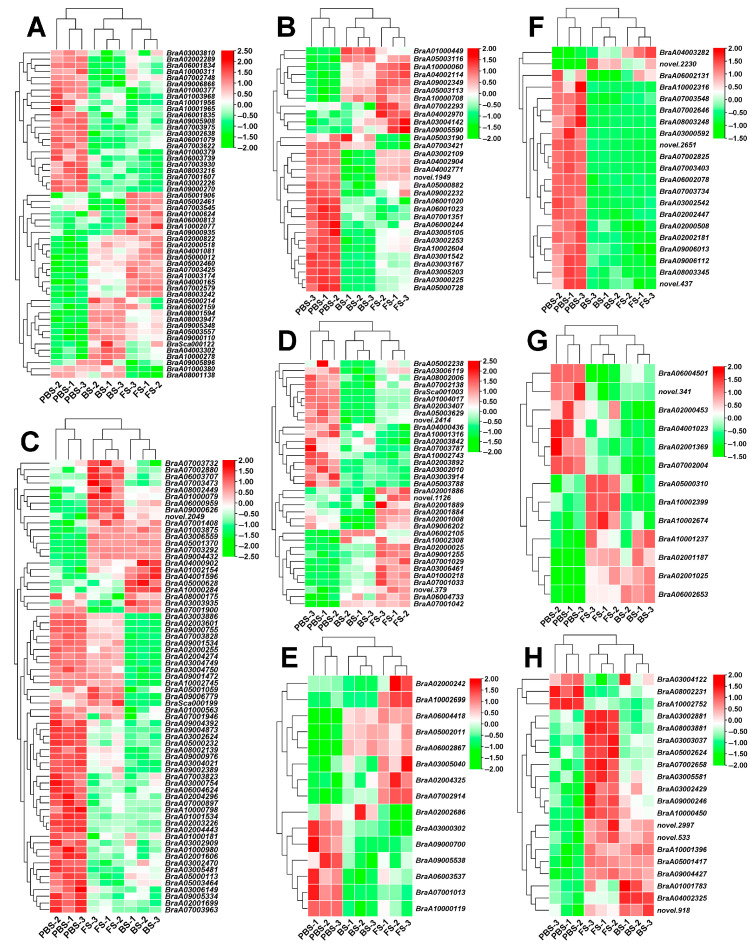
Heatmap analysis of the expression profiles of DEGs involved in plant hormone signal transduction. (**A**) Auxin signaling pathway. (**B**) ABA signaling pathway. (**C**) ET signaling pathway. (**D**) BR signaling pathway. (**E**) GA signaling pathway. (**F**) JA signaling pathway. (**G**) CK signaling pathway. (**H**) SA signaling pathway. Red represents upregulated genes; green represents downregulated genes. PBS, BS, and FS represent the pre-bolting stage, bolting stage, and flowering stage, respectively.

Floral transition is modulated by intricate hormonal signaling networks. To investigate the molecular mechanisms, the dynamic expression patterns of genes involved in major hormone signaling pathways, auxin, abscisic acid (ABA), ethylene (ET), brassinosteroids (BR), gibberellins (GA), jasmonic acid (JA), cytokinins (CK), and salicylic acid (SA), were analyzed. In the auxin signaling pathway, both up- and downregulated genes were identified, with the *auxin-responsive protein SAUR21* (*BrSAUR21*, *BraA02000822*) demonstrating significant upregulation (Figure 5A). The ABA signaling pathway exhibited a differential regulation of key genes, with *ABA RECEPTOR PYR1-LIKE 6* (*BrPYL6*, *BraA05000728*) showing significant downregulation while other DEGs were markedly upregulated during BS and FS (Figure 5B). Moreover, a transcriptional analysis of ET signaling showed stage-specific expression, with genes like *ETHYLENE RESPONSE SENSOR 1* (*BrERS1*, *BraA05000113*) showing significant downregulation during pre-floral stages. However, critical transcriptional regulators including *ETHYLENE-RESPONSIVE TRANSCRIPTION FACTOR 11* (*BrERF11*, *BraA09004432*) showed a marked upregulation during post-floral development (Figure 5C). The BR pathway gene *BRASSINAZOLE-RESISTANT 2* (*BrBZR2*, *BraA06002105*) was significantly upregulated in BS, indicating increased BR pathway activation during floral transition (Figure 5D).

Further, the GA biosynthetic gene *GIBBERELLIN 20 OXIDASE 2* (*BrGA20ox2*, *BraA03005040*) exhibited a significant upregulation during floral transition, indicating its pivotal role in increasing the synthesis of bioactive GAs that activate downstream flowering signals (Figure 5E). Furthermore, most JA pathway genes were significantly downregulated during floral transition, exemplified by the repression of the *JASMONATE ZIM DOMAIN-CONTAINING PROTEIN* (*BrJAZ10*, *BraA03000592*), suggesting a shift away from JA-mediated defense responses in favor of reproductive growth processes (Figure 5F). An analysis of CK signaling genes showed differential regulation, with *CYTOKININ DEHYDROGENASE 3* (*BrCKX3*, *BraA02001369*) significantly downregulated, while *LONELY GUY 8* (*BrLOG8*, *BraA10002399*) was significantly upregulated (Figure 5G). Moreover, key components of the SA biosynthesis and signaling pathway were predominantly upregulated, particularly *ISOCHORISMATE SYNTHASE 1* (*BrICS1*, *BraA07002658*), indicating a possible activation of SA-mediated pathways during the floral transition (Figure 5H).

**Figure 6:**
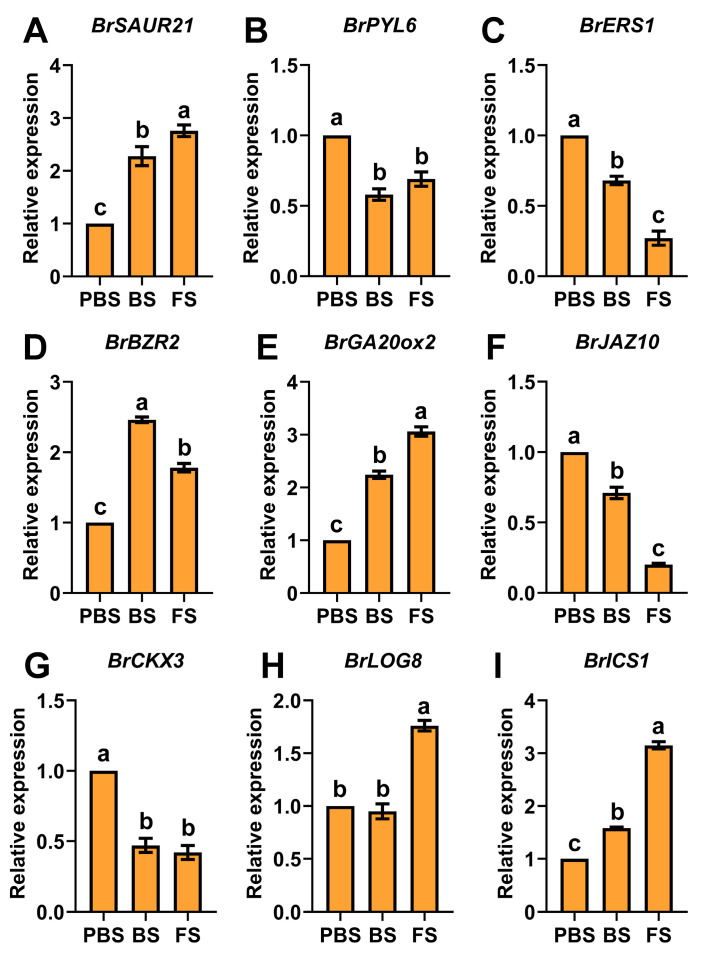
qRT-PCR validation of DEGs involved in plant hormone signal transduction. Relative expression of (**A**) *BrSAUR21*, (**B**) *BrPYL6*, (**C**) *BrERS1*, (**D**) *BrBZR2*, (**E**) *BrGA20ox2*, (**F**) *BrJAZ10*, (**G**) *BrCKX3*, (**H**) *BrLOG8*, and (**I**) *BrICS1*. Different lowercase letters denote statistically significant differences (*p* < 0.05, one-way ANOVA). PBS, BS, and FS represent the pre-bolting stage, bolting stage, and flowering stage, respectively.

Furthermore, the transcript abundance profiles of *BrSAUR21*, *BrPYL6*, *BrERS1*, *BrBZR2*, *BrGA20ox2*, *BrJAZ10*, *BrCKX3*, *BrLOG8*, and *BrICS1* were verified by qRT-PCR, with expression trends aligning closely with those observed in transcriptome sequencing during floral transition (Figure 6A–I). These results confirm that floral transition in Chinese cabbage is modulated by well-coordinated hormonal crosstalk, where each phytohormone signaling pathway displays specific temporal expression patterns that facilitate the vegetative-to-reproductive developmental switch.

### 2.5. Expression Patterns of DEGs in MAPK Signaling Pathway

**Figure 7:**
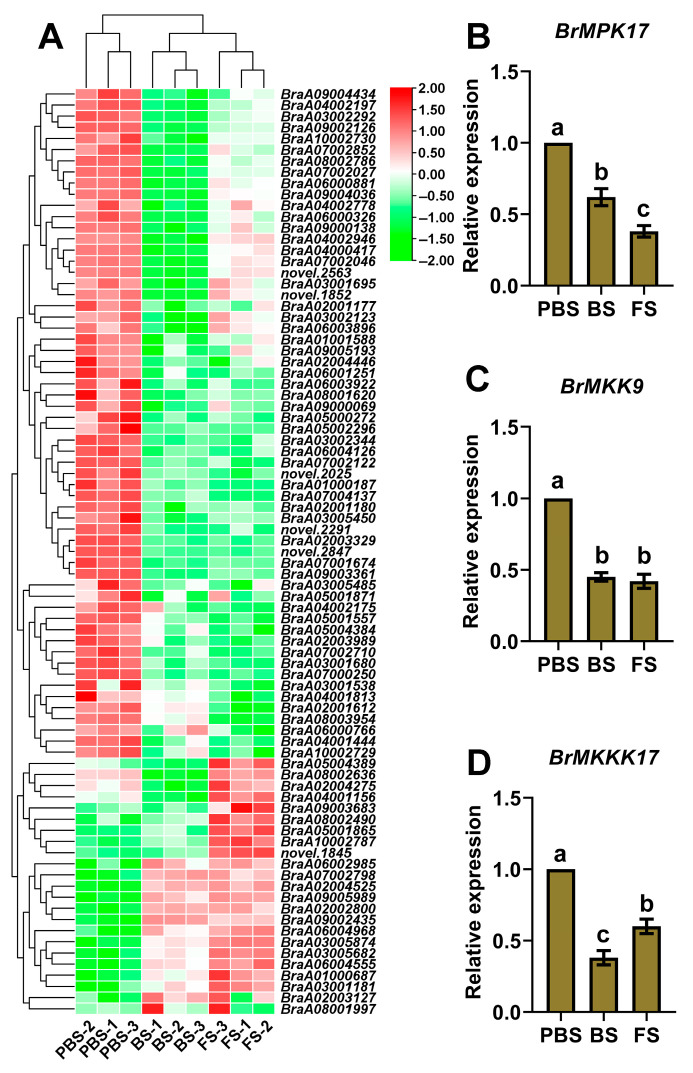
Expression patterns of DEGs in the MAPK signaling pathway. (**A**) Heatmap analysis. Each column denotes an independent biological sample, while each row represents a unique gene transcript. Red represents upregulated genes; green represents downregulated genes. (**B**–**D**) qRT-PCR validation. Different lowercase letters denote statistically significant differences (*p* < 0.05, one-way ANOVA). PBS, BS, and FS represent the pre-bolting stage, bolting stage, and flowering stage, respectively.

The MAPK signaling pathway is a critical molecular interface that supports the integration of internal and external cues to regulate the floral transition. Therefore, the expression patterns of DEGs associated with the MAPK signaling pathway during the floral transition in Chinese cabbage were analyzed. A transcriptome analysis revealed pronounced stage-specific expression patterns of the majority of MAPK-related genes, showing peak transcript levels during PBS followed by a marked downregulation at BS and FS stages (Figure 7A). The coordinated downregulation of these pathway components, such as *MITOGEN-ACTIVATED PROTEIN KINASE 17* (*BrMPK17*, *BraA02003329*), *MITOGEN-ACTIVATED PROTEIN KINASE KINASE 9* (*BrMKK9*, *BraA07002710*), and *MITOGEN-ACTIVATED PROTEIN KINASE KINASE KINASE* (*BrMKKK17*, *BraA09004434*), may indicate a key regulatory mechanism in controlling the shift from vegetative growth to reproductive development. Furthermore, the transcriptome sequencing results were highly consistent with the expression patterns of *BrMPK17*, *BrMKK9*, and *BrMKKK17*, as demonstrated by qRT-PCR validation (Figure 7B–D).

### 2.6. Expression Profiling of DEGs in the OGs Pathway

**Figure 8:**
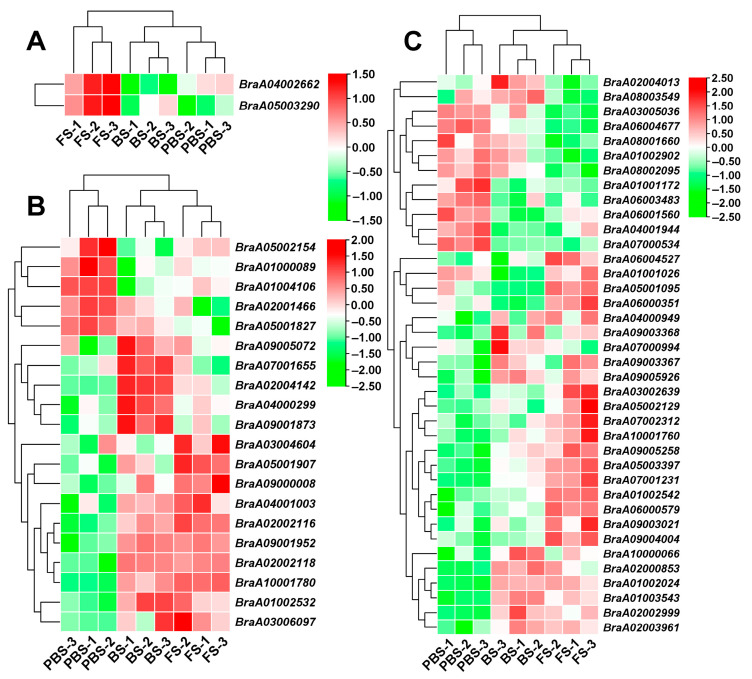
Heatmap analysis of the expression profiles of DEGs involved in the OGs pathway. (**A**) BrOGs signaling pathway. (**B**) BSGs signaling pathway. (**C**) CSGs signaling pathway. Red represents upregulated genes; green represents downregulated genes. PBS, BS, and FS represent the pre-bolting stage, bolting stage, and flowering stage, respectively.

**Figure 9:**
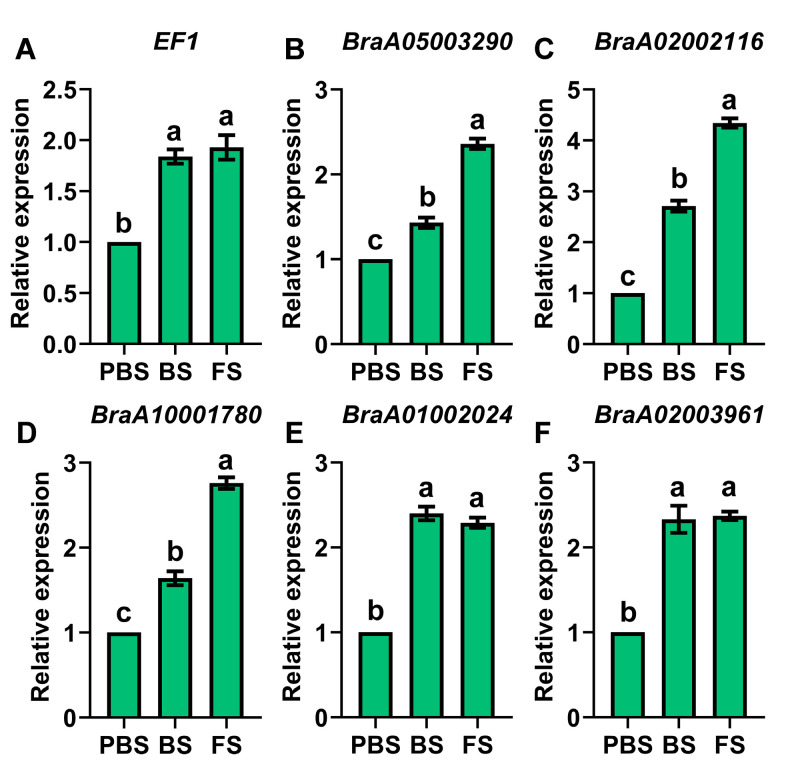
qRT-PCR validation of DEGs involved in the OGs pathway. Relative expression of (**A**) *EF1*, (**B**) *BraA05003290*, (**C**) *BraA02002116*, (**D**) *BraA10001780*, (**E**) *BraA01002024*, and (**F**) *BraA02003961*. Different lowercase letters denote statistically significant differences (*p* < 0.05, one-way ANOVA). PBS, BS, and FS represent the pre-bolting stage, bolting stage, and flowering stage, respectively.

The OGs pathway indicates a novel floral induction mechanism that regulates the flowering process. Previous studies identified and characterized BrOGs, *Brassica*-specific genes (BSGs), and Cruciferae-specific genes (CSGs) in *B. rapa* [2,32]. Based on these findings, current transcriptome analyses identified differential expression of significant DEGs in the OGs pathway, comprising 2 BrOGs, 20 BSGs, and 38 CSGs, indicating potential functions in the control of flowering (Figure 8). During FS, there was a considerable increase in two BrOGs: *BraA04002662* (*EARLY FLOWERING 1*, *EF1*) and *BraA05003290* (Figure 8A). Most BSGs showed significant upregulation during both BS and FS relative to the PBS, such as *BraA02002116* and *BraA10001780* (Figure 8B). Likewise, the majority of CSGs exhibited substantial transcriptional activity during floral transition, with significant upregulation in both BS and FS compared to PBS, highlighted by *BraA01002024* and *BraA02003961* (Figure 8C). The relative expression patterns of *EF1*, *BraA05003290*, *BraA02002116*, *BraA10001780*, *BraA01002024*, and *BraA02003961* were also consistently associated with transcriptome sequencing data, based on qRT-PCR validation (Figure 9A–F). These findings reveal that the coordinated upregulation of the evolutionarily distinct gene groups, BrOGs, BSGs, and CSGs, within the OGs pathway orchestrates floral transition in Chinese cabbage. This demonstrates a novel regulatory mechanism in which OGs function in conjunction with conserved flowering regulators to control floral transition.

### 2.7. Orphan Gene EARLY FLOWERING 1 Positively Regulates Floral Initiation

**Figure 10:**
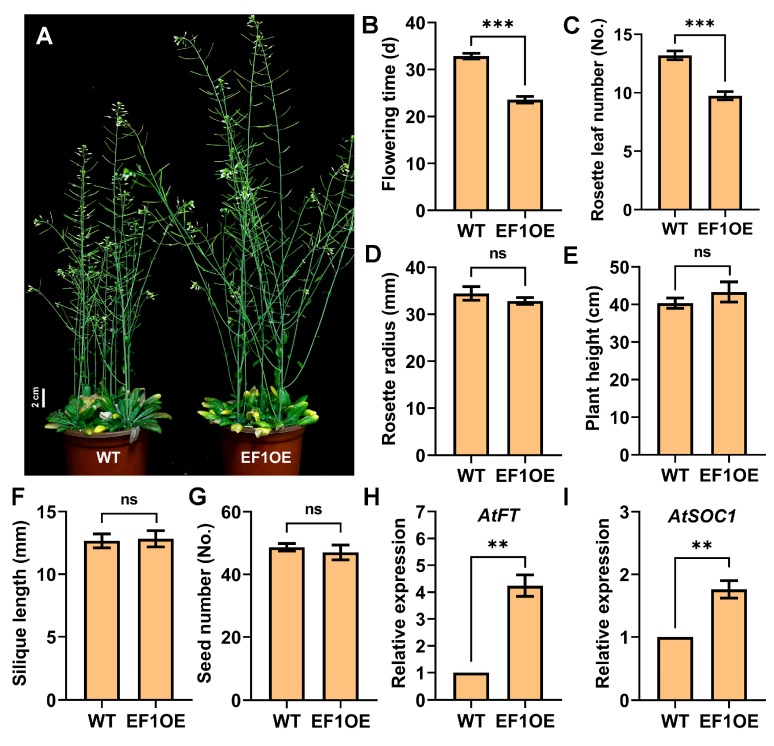
Phenotypic characterization of *Arabidopsis* EF1OE lines. (**A**) Early flowering phenotype of EF1OE transgenic plants. Scale bar = 2 cm. (**B**) Flowering time. (**C**) Rosette leaf number. (**D**) Rosette radius. (**E**) Plant height. (**F**) Silique length. (**G**) Seed number. (**H**,**I**) Expression analysis of *AtFT* and *AtSOC1*. Data are means ± SE of three independent measurements. Significant differences were identified when comparing WT and EF1OE plants using Student’s *t*-tests (ns, *p* > 0.05, ** *p* < 0.01, *** *p* < 0.001).

Previous studies constructed a BrOGs overexpression (BrOGsOE) library in *Arabidopsis* to facilitate functional characterization of these genes [2]. Among them, *EF1* overexpression (EF1OE) lines exhibited a clear early flowering phenotype, with transgenic plants flowering significantly earlier than wild-type (WT) controls under long-day (LD) conditions (Figure 10A,B). Compared to WT, EF1OE lines produced fewer rosette leaves (Figure 10C). However, there were no statistically significant changes between EF1OE lines and WT plants in terms of rosette radius, plant height, silique length, and seed quantity (Figure 10D–G). Moreover, the expression levels of *AtFT* and *AtSOC1* were significantly upregulated in EF1OE lines compared with WT plants (Figure 10H,I). The results support the role of *EF1* in selectively regulating floral transition, suggesting its potential as a candidate gene for breeding bolting-resistant Chinese cabbage.

## 3. Discussion

The floral transition is a critical developmental transition in the plant life cycle, indicating an irreversible transformation from vegetative growth to reproductive development. This transition process serves as a critical determinant of both crop yield potential and reproductive success in plants [36]. This process is regulated by a complex interplay of genetic, molecular, and environmental cues [20]. In the present study, temporal transcriptome profiling of Chinese cabbage was performed to investigate the regulatory basis of floral transition. The results demonstrate stage-specific gene expression changes and highlight the roles of hormonal interactions, MAPK signaling, and the OGs pathway in modulating the flowering time.

Using transcriptomic analysis across PBS, BS, and FS stages, 7092 DEGs with distinct temporal expression profiles were identified, with the transition from bolting to flowering (FS_vs_BS) exhibiting the most substantial transcriptomic changes. A recent study reported a greater number of DEGs between early- and late-bolting Chinese cabbage floral buds [37], likely attributable to the tissue-specific nature of transcriptome profiling, as the current leaf-based analysis captured early transition events, whereas bud-focused studies reflect later-stage organogenesis involving broader gene expression shifts. The precise timing of floral initiation is maintained by a tightly regulated antagonistic mechanism, as demonstrated by the downregulation of flowering repressors (*BrFLM* and *BrAP2*) and the simultaneous upregulation of flowering activators (*BrFD*, *BrFT*, and *BrSOC1s*). This is consistent with previous investigations conducted on *Arabidopsis* and *Brassica* species, which have shown that the balance between floral repressors and activators is a determining factor in flowering competence [27,38,39,40,41,42]. Therefore, identifying these key regulators presents valuable molecular targets for developing bolt-resistant Chinese cabbage cultivars. Building on these findings, future studies will incorporate multi-organ transcriptome profiling encompassing shoot apical meristems, floral buds, and developing reproductive organs to provide a more comprehensive understanding of spatiotemporal gene regulation throughout the entire floral transition process, from early induction to late organogenesis stages.

Plant hormones act as central integrators of internal and external cues to regulate the flowering time [43]. Our findings uncover distinct temporal dynamics in the expression of hormone-responsive genes across different developmental stages. In the GA pathway, the upregulation of *BrGA20ox2* suggests enhanced GA biosynthesis, consistent with its established function in promoting bolting and flowering [44]. Similarly, the upregulation of *BrSAUR21* and *BrBZR2* in the auxin and BR pathways suggests their involvement in promoting floral transition, potentially through modulation of growth. Previous studies have emphasized auxin’s critical function in floral development, highlighting its role in restricting stem cell pluripotency at the shoot apical meristem and activating downstream genetic programs that initiate floral organ primordia, therefore contributing directly to floral induction [45,46]. Moreover, the floral transition is regulated by BR signaling through the integration of endogenous and environmental inputs, particularly via modulation of *FT* expression and coordination with other flowering pathways [47]. Furthermore, ABA-related DEGs demonstrated both positive and negative regulators of flowering, including *BrPYL6*, consistent with previous studies showing ABA’s dual regulatory role during flowering [43]. In JA signaling, a downregulation of DEGs such as *BrJAZ10* suggests suppression of defense-related pathways, enabling a developmental shift toward reproductive development. This pattern reflects a suppression of JA-mediated inhibitory effects to facilitate floral initiation, which aligns with previous reports [48]. Further, ET pathway genes showed stage-specific dynamics, with an early repression of *BrERS1* and a later upregulation of *BrERF11*, suggesting ET may delay initial floral transition but later support the floral organ development. Previous reports confirm that ET functions in a complex, context-dependent manner, as demonstrated by divergent flowering responses in mutants with modified ET biosynthesis or signaling pathways [43]. The downregulation of *BrCKX3* alongside the upregulation of *BrLOG8* suggests that CK signaling may support floral meristem activity, consistent with findings from previous studies [49]. Within the SA signaling pathway, the induction of *BrICS1* indicates a potential role for SA in flowering, possibly connecting defense mechanisms to reproductive timing. This observation is consistent with reports that SA accelerates flowering [43]. These results demonstrate that a complex hormonal network regulates floral transition in Chinese cabbage, wherein multiple phytohormonal pathways exhibit tightly coordinated yet temporally distinct expression patterns to regulate the shift from vegetative to reproductive development. This coordinated hormonal interplay supports a flexible yet precise flowering mechanism that balances endogenous and environmental signals to optimize flowering timing.

While the distinct roles of individual hormones in floral transition are well documented, emerging evidence highlights the importance of hormonal crosstalk in fine-tuning the flowering time [43]. For instance, GA and JA antagonistically regulate the flowering time, with GA as an inducer and JA as an inhibitor, while JA also interacts with GA and other phytohormones like ABA, ET, SA, and auxin to modulate various physiological processes [48]. Future studies should explore how these hormonal pathways integrate at the molecular level. Such investigations would provide deeper insights into the dynamic hormonal coordination underlying floral transition.

Various cellular processes are regulated by MAPK cascades, critical signaling modules associated with hormonal responses and downstream of receptors that sense endogenous stimuli or exogenous cues [50,51]. The current analysis reveals dynamic expression patterns of MAPK pathway components throughout the floral transition in Chinese cabbage. The majority of MAPK pathway genes exhibit increased expression levels during PBS, which decrease substantially during BS and FS. The results suggest that MAPK signaling may act as a negative regulator or be actively modulated to facilitate the transition to reproductive development, as indicated by the suppression of *BrMPK17*, *BrMKK9*, and *BrMKKK17* (Figure 7). Previous studies have shown that MAPK pathways impact the flowering time via complex interactions with hormone signaling and floral regulatory networks, and may also affect other flowering-related physiological processes [52]. The coordinated downregulation of multiple MAPK components implies a potential systemic suppression of the pathway, which may represent a regulatory mechanism to avoid interference with critical floral induction signals. To further understand the role of MAPK in floral transition, future studies should investigate downstream targets (e.g., flowering-related transcription factors) and upstream regulators (e.g., photoperiod, temperature, or hormones).

A significant outcome of this study is the identification of OGs as contributors to floral transition. Transcriptomic analysis revealed that genes from BrOGs, BSGs, and CSGs were consistently upregulated during BS and FS, suggesting their coordinated involvement in reproductive development. These lineage-specific genes may have evolved regulatory functions that fine-tune the flowering time in *B. rapa*, potentially increasing species adaptation and diversification. Previous studies have highlighted the regulatory significance of OGs in flowering [2,5]. Although the precise processes are yet to be identified, functional validation in *Arabidopsis* further showed that *EF1* overexpression accelerates flowering. To advance our understanding of the molecular mechanisms, future investigations should integrate systematic identification of upstream TFs controlling *EF1* expression via promoter analysis and yeast one-hybrid screening with in-depth characterization of EF1 protein interaction networks, which will elucidate whether its regulation of the flowering time occurs through evolutionarily conserved pathways or species-specific regulatory networks. This finding broadens current knowledge of flowering regulation by revealing mechanisms beyond conserved genetic pathways and underscores the potential application of OGs in crop improvement.

This study establishes a comprehensive molecular framework for floral transition in Chinese cabbage by integrating transcriptomic profiles, hormonal signaling, and the functions of novel genes. Floral induction timing appears to be regulated by the antagonistic interaction between floral repressors and activators, modulated through hormone-mediated signaling crosstalk. The suppression of MAPK signaling components may support flowering by downregulating stress-associated pathways. Moreover, OGs, particularly *EF1*, emerge as novel regulators with potential utility in breeding programs targeting bolting resistance. Further research studies should aim to validate candidate genes such as *EF1* using CRISPR/Cas9-based knockout or overexpression systems in Chinese cabbage. Moreover, investigations into epigenetic landscapes and post-transcriptional networks of flowering genes may provide further mechanistic insights. These findings advance the current understanding of the floral transition process and offer new molecular targets for breeding early- or late-flowering varieties to optimize yield and stress resilience in *Brassica* crops.

## 4. Materials and Methods

### 4.1. Plant Materials

Seeds of the germinated Chinese cabbage inbred line ‘CC026’ were cultivated at 4 °C for 4 weeks before they were transferred into nutrient pots. Leaf samples were harvested at three developmental stages: five days before bolting (pre-bolting stage, PBS), at bolting (BS), and during flowering (FS), for downstream transcriptome sequencing analysis. Three biological replicates were obtained for each stage, with each replicate comprising three plants; samples were immediately flash-frozen in liquid nitrogen and stored at −80 °C until subsequent analysis. Wild-type (WT) *Arabidopsis* Columbia-0 (Col-0) and transgenic lines were grown under conditions established in previous studies [2].

### 4.2. Total RNA Isolation, Library Preparation, Sequencing, and Transcriptome Assembly

RNA extraction, library preparation, sequencing, and transcriptome assembly were performed using established methods [53]. An Illumina HiSeq system (Illumina Inc., San Diego, CA, USA) was used for sequencing, yielding paired-end reads of 125/150 bp. Fastp (v0.23.2) was used to implement data quality control. Using HISAT2 (v2.2.1), clean reads were mapped to the *B. rapa* v2.5 genome that is accessible via the *Brassica* Database (BRAD) (http://brassicadb.cn/#/) (accessed on 16 November 2024).

### 4.3. Functional Characterization and Novel Gene Identification

The functional characterization of genes was performed using BLAST-based (v2.16.0) alignment against established reference databases, with Gene Ontology (GO) applied for functional classification and the Kyoto Encyclopedia of Genes and Genomes (KEGG) used for pathway mapping. The identification of novel genes was performed using StringTie (v2.1.6).

### 4.4. Gene Expression Quantification, Differential Gene Expression Profiling, and Enrichment Analysis

Transcript abundance was assessed as fragments per kilobase of exon per million mapped reads (FPKM), adjusting for gene length and sequencing depth. Differential expression analysis was performed using DESeq2 (v1.22.1) with stringent thresholds of |log2 fold change| ≥ 1 and false discovery rate (FDR) < 0.05. Genes satisfying both thresholds were classified as differentially expressed genes (DEGs). A classification of GO and KEGG enrichment analysis was conducted based on previously reported methods [53].

### 4.5. Generation and Characterization of EF1-Overexpressing Transgenic Arabidopsis Plants

The vector construction, transformation of *Arabidopsis*, and selection of transgenic plants were conducted according to previously described protocols [2]. The *EF1*-overexpression vector was constructed using primer pairs provided in Table S4. A phenotypic evaluation of T_3_ generation transgenic *Arabidopsis* plants was performed as detailed in the previous study [2].

### 4.6. Quantitative Real-Time PCR Validation

Total RNA was extracted, reverse transcribed into cDNA, and analyzed by quantitative real-time PCR (qRT-PCR) using methods described in previous studies [5]. Samples from EF1OE and WT plants grown for 20 d under LD conditions were collected to validate *AtFT* and *AtSOC1* expression, with three biological replicates and at least three plants per replicate. *BrEF-1-α* and *AtActin* were selected as the internal control gene in Chinese cabbage and *Arabidopsis*, respectively. A relative quantification of gene expression was performed using the 2^−ΔΔCT^ method [54]. The experiment was conducted with three independent biological replicates. Details of primers used are provided in Table S4.

### 4.7. Statistical Analysis

All statistical analyses were performed using SPSS v19.0. Student’s *t*-test was applied for comparisons between two groups, while one-way ANOVA followed by Duncan’s multiple range test was used for multiple group comparisons. Data visualization was carried out using GraphPad Prism v8.0.2 and TBtools-II v2.310 [55].

## 5. Conclusions

The transcriptomic atlas generated in this study provides a comprehensive molecular characterization of floral transition in Chinese cabbage, highlighting dynamic transcriptional changes and coordinated hormonal signaling throughout reproductive development. Analysis revealed 7092 DEGs with developmental stage-specific expression, including core regulators of flowering and components of multiple hormonal pathways. The downregulation of MAPK signaling genes and upregulation of OGs such as *EF1* indicate their distinct regulatory functions in floral initiation. The *EF1* overexpression in *Arabidopsis* accelerated flowering, suggesting potential use in breeding bolting-resistant cultivars. These findings advance the current understanding of floral transition mechanisms in *Brassica* species and emphasize the integration of conserved and lineage-specific pathways in reproductive regulation.

## Data Availability

The raw data supporting the conclusions of this article will be made available by the authors on request.

## Supplementary Materials

The following supporting information can be downloaded at: https://www.mdpi.com/article/10.3390/plants14142236/s1, Table S1. Transcriptome sequencing data. Table S2. Transcriptome sequencing reads alignment with reference genome data. Table S3. Expression patterns and annotations of 81 core flowering pathway DEGs. Table S4. Primers used in this study.

## Abbreviations

The following abbreviations are used in this manuscript:

ABA	Abscisic acid
BP	Biological process
BR	Brassinosteroids
*BrOGs*	*Brassica rapa* orphan genes
BrOGsOE	BrOGs overexpression
BS	Bolting stage
BSGs	Brassica-specific genes
CC	Cellular component
CK	Cytokinins
CSGs	Cruciferae-specific genes
DEGs	Differentially expressed genes
*EF1*	*EARLY FLOWERING 1*
EF1OE	*EF1* overexpression
ET	Ethylene
FPKM	Fragments per kilobase of exon per million mapped reads
FS	Flowering stage
GA	Gibberellin
GO	Gene Ontology
JA	Jasmonic acid
KEGG	Kyoto Encyclopedia of Genes and Genomes
MAPK	MITOGEN-ACTIVATED PROTEIN KINASE
MF	Molecular function
OE	Overexpression
OGs	Orphan genes
PBS	Pre-bolting stage
PCA	Principal component analysis
qRT-PCR	quantitative Real-Time PCR
SA	Salicylic acid
WT	Wild-type
